# Mutations in the Esx-3 secretion system confer resistance to multiple chemical scaffolds in Mycobacterium tuberculosis

**DOI:** 10.1099/mic.0.001625

**Published:** 2025-11-11

**Authors:** Theresa O’Malley, Matthew B. McNeil, Devon Dennison, Thomas Ioerger, Tanya Parish

**Affiliations:** 1 TB Discovery Research, Infectious Disease Research Institute, Seattle, WA, USA; 2 Department of Computer Science and Engineering, Texas A&M University, College Station, TX, USA; 3 Center for Global Infectious Disease Research, Seattle Children’s Research Institute, Seattle, WA 98101, USA; 4 Department of Pediatrics, University of Washington School of Medicine, Seattle, WA 98195, USA

**Keywords:** antibiotic resistance, metal ion homeostasis, mycobacteria, tuberculosis

## Abstract

We determined the mechanism of resistance to seven chemical series with potent activity against *Mycobacterium tuberculosis*. Resistant mutants were isolated against the aminothiazoles, phenylhydrazones, 8-hydroxyquinolines, nitazoxanides, phenyl alkylimidazoles, morpholino thiophenes and trifluoromethyl pyrimidinones. We demonstrated that mutations in several components of the Esx-3 type VII secretion system (EccA3, EccB3, EccC3 and EccD3) conferred resistance to these disparate scaffolds. We conclude that mutations in Esx-3 are a common mechanism of resistance to anti-tubercular agents, which may have clinical relevance for new drugs.

## Data Availability

Sequences of strains are available in Table S1 (available in the online Supplementary Material).

## Introduction

*Mycobacterium tuberculosis* is a pathogen of global importance and the causative agent of more than 1 million deaths annually [1,2]. A concerted effort to discover new drugs has led to the identification of numerous chemical scaffolds with potent anti-tubercular activity.

## Results and discussion

**Fig. 1.**
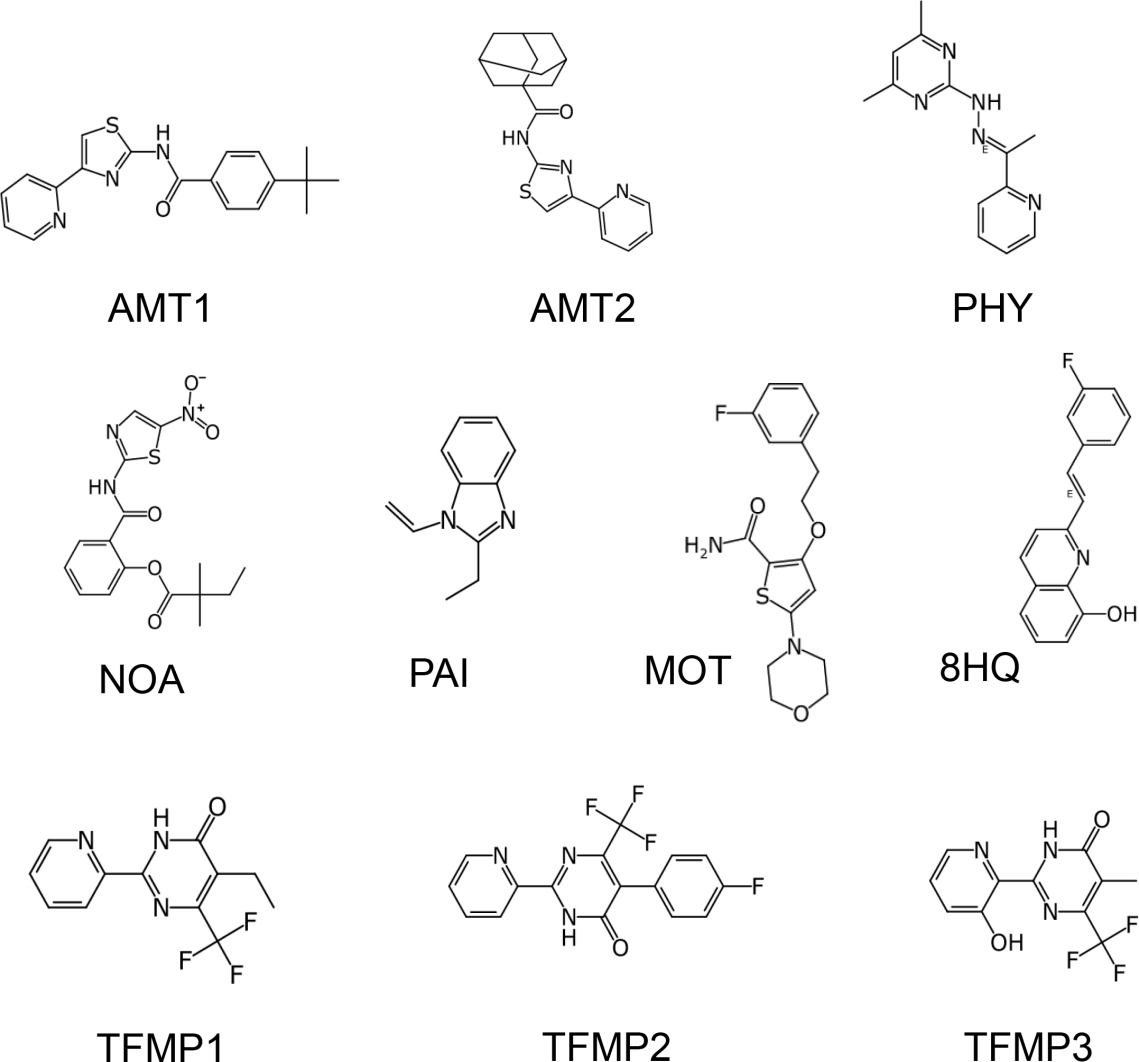
Structure of molecules used in this study.

As part of a thorough evaluation of novel chemical series, we were interested in determining the mechanism of resistance to novel agents. We selected representative molecules from several series we have previously explored (Fig. 1); these include the aminothiazoles (AMT) [3], phenylhydrazones (PHY) [4,5], 8-hydroxyquinolines (8HQ) [6], nitazoxanides (NOA) [7], phenyl alkylimidazoles (PAI) [8,9], morpholino thiophenes (MOT) [10] and trifluoromethyl pyrimidinones (TFMP) [11]. We isolated resistant mutants of *M. tuberculosis* as previously described [12]. Briefly, we determined the MIC of each molecule against *M. tuberculosis* grown on Middlebrook 7H10 agar medium supplemented with 10% (v/v) OADC (oleic acid, bovine serum albumin, d-glucose, catalase; Becton Dickinson). The MIC was defined as the minimum concentration required to reduce c.f.u. by 99% [13]. We then plated 10^7^ to 10^9^ c.f.u. onto agar plates containing 5× or 10× MIC and cultured them for 3–6 weeks. Individual colonies were isolated and tested for resistance on solid medium.

We obtained resistant mutants for each chemical scaffold (Table 1). The level of resistance varied from low-level (up to fourfold resistance) to high-level resistance (up to 125-fold resistance). We selected a small set of resistant isolates for whole-genome sequencing. Genomic DNA was prepared and sequenced as previously described [12](Table S1) . Mutations were verified by PCR amplification and sequencing. We analysed the genomic data to identify SNPs. For each series, we looked for genetic variants (including SNPs and insertion/deletions) genome-wide, excluding PPE/PGRS genes and poorly sequenced regions (with coverage <10× or base-call purity <70%). Interestingly, we found mutations in the type VII secretion system Esx-3 in resistant isolates for all seven series; strains with mutations in Esx-3 are reported in Table 1 (all mutations in each strain are detailed). For some molecules, we found mutations in other genes in other strains (not included in the Table or reported here), suggesting that there are multiple mechanisms of resistance or targets, but here we focused on the strains with mutations in Esx-3.

**Table 1.** Mutations in *M. tuberculosis*-resistant isolates

Strain	Series	MIC (µM)		Fold-change	Mutation
		WT	Resistant strain		
LP-0106963-RM1	AMT1	0.125	0.5	4	EccB3 N26 deletion
LP-0106963-RM2	AMT1	0.125	0.5	4	EccD3 G450R
LP-0106963-RM3	AMT1	0.125	0.5	4	EccB3 N26 deletion
LP-0106967-RM5	AMT2	0.25	2.5	10	EccC3 D113Y
LP-0107828-RM1	PHY	3.1	100	32	EccB3 R14L
LP-0107828-RM5	PHY	3.1	100	32	EccB3 R14L, SugI E231D
LP-0107828-RM6	PHY	3.1	50	16	EccB3 R14L, SugI E231D
LP-0257184-RM3	8HQ	0.5	2.5	5	EccD3 G450R
LP-0257184-RM4	8HQ	0.5	5	10	EccA3 T389I, SigA T365A
LP-0257184-RM5	8HQ	0.5	1.25	3	EccB3 T21A
LP-0257184-RM6	8HQ	0.5	1.25	3	EccD3 G450R
LP-0261867-RM1	NOA	0.5	10	20	EccC3 L77P
LP-0261867-RM5	NOA	0.5	10	20	EccC3 E25D
LP-0261867-RM7	NOA	0.5	10	20	EccC3 L167P
LP-0333226-RM2	PAI	10	100	10	EccD3 L312Q, Rv1782 R39C
LP-0333226-RM3	PAI	10	100	10	EccA3 T389I, Rv1782 R39C, Ltp3 A70V
LP-0474076-RM1	MOT	0.8	100	125	EccA3 E237K
LP-0474076-RM2	MOT	0.8	100	125	EccC3 V185L
LP-0474076-RM3	MOT	0.8	100	125	EccC3 L167P
LP-0497747-RM1	TFMP1	10	>20	>2	EccD3 E32K
LP-0504061-RM2	TFMP2	1.6	25	16	EccC3 D181N
LP-0504069-RM2	TFMP3	<0.8	12.5	>16	EccC3 D181N

MICs were determined on solid agar. Number relates to structures in Fig. 1.

For the aminothiazole series, we observed mutations in EccB3 (3 bp deletion of a single amino acid) or EccD3 (G450R or D133Y), which conferred low-level resistance with a fourfold change in solid MIC. For the hydrazone series, we observed high-level resistance (16-fold to 32-fold) resulting from a single mutation in EccB3 (R14L), which was accompanied by a mutation in SugI (E231D) in two of the three strains. For the 8HQ, we observed a variable level of resistance, with low-level resistance from EccB3_T21A_ or EccD3_G450R_, but a higher level of resistance (10-fold) from EccA3_T389I_. High-level resistance (20-fold) to the nitazoxanide compounds resulted from mutations in EccC3 (L77P, E25D or L167P). Resistance to PAI was conferred by EccD3_L312Q_ or EccA3_T389I_; in this case, both strains had a mutation in another gene, Rv1782_R39C_, and one strain had an additional mutation in Ltp3 (A70V). Mutations in EccA3 (E237K) or EccC3 (V185L or L167P) conferred high-level resistance to MOT (125-fold). Finally, a mutation in EccC3 (D181N) conferred high-level resistance (>16-fold) to TFMP, with EccD_E32K_ conferring low-level resistance.

Esx-3 encodes one of five type VII secretion systems but is the only one essential for viability under standard growth conditions [14]. EccA3 encodes a cytoplasmic ATPase, and EccB3/C3/D3 encode membrane-associated proteins that form part of the secretion complex in the cytoplasmic membrane. The Esx-3 system plays a key role in iron uptake in all mycobacteria, and its expression is controlled by iron and zinc in *M. tuberculosis* [14]. The system becomes dispensable when iron is supplied in excess, suggesting that its essentiality is related to iron uptake [14]. The Esx-3 machinery exports a number of proteins, including EsxG/EsxH, which are required for virulence; PE5/PPE4, which are required for siderophore-mediated iron uptake; and PE15/PPE20, which are required for calcium transport. These data suggest that the system plays a crucial role in metal ion homeostasis beyond iron uptake. Notably, we found many different mutations in several components of the Esx-3 system; thus, we observed mutations in EccA3, EccB3, EccC3 and EccD3. We mapped these mutations onto the structure of the *Mycobacterium smegmatis* complex (PBD 6LAR) [15]. Mutations were located within (for EccB3 and EccC3) or close to the cytoplasmic domain (for EccD3). Since the mutations were in several components of the system, we thought it is unlikely that Esx-3 is the target of these disparate series, and this was a resistance mechanism.

Recent work has suggested that ivermectin inhibits the growth of *M. smegmatis* via inhibition of EccD3 [16]. However, knockdown of EccD3 had no impact on the growth of *M. smegmatis* and led to an increased potency (MIC) of ivermectin of only twofold, even though gene transcripts were reduced >90-fold [16]. This is not surprising, since Esx-3 is not essential *in M. smegmatis* under standard culture conditions. Since the MIC for linezolid was also reduced twofold, this suggests that ivermectin does not inhibit growth by targeting EccD3, but that downregulation of the Esx-3 system has a non-specific effect on antibiotic sensitivity in the non-pathogenic species. Whether this also occurs in *M. tuberculosis* remains unknown.

Many of the mutations we identified are likely to cause large protein perturbations, for example, by incorporating proline [17], as in the EccC3 L77P and L167P mutations, or by changing charged amino acids such as the loss of aspartate or glutamate in EccC3_D113Y_, EccC3_D1841N_ and EccD3 _E32K._ Previous work demonstrated that mutations in Esx-3 led to resistance to a novel iron chelator, suggesting that increased iron uptake was occurring. This would argue against the hypothesis that these mutations reduce Esx activity, but we do not yet know the downstream consequences of the mutations on secretion of individual components [18]. In addition, since all of our strains were viable in standard medium, we do not believe that the mutations lead to a complete loss of function. We do not know how these mutations lead to resistance to such a wide range of chemical scaffolds, but it is interesting that the 8HQ are copper ionophores [19]. Since copper toxicity can be reversed by adding iron, this supports the hypothesis that metal-ion homeostasis is the key process in determining susceptibility/resistance, and that mutations in Esx-3 could lead to increased iron uptake.

It is interesting that we observed resistance to two series known to target QcrB. QcrB is part of the cytochrome bcc–aa3 supercomplex and forms a part of the electron transport chain [20]. High-level resistance was conferred to both PAI and MOT by mutations in EccA3, EccC3 and EccD3. We have previously demonstrated that mutations in QcrB also lead to resistance, and that molecules deplete ATP, consistent with their mode of action in targeting the electron transport chain [9,10]. We propose that disruption of metal-ion homeostasis can also impact cytochrome oxidase activity, since electron transfer is dependent on both copper and iron–sulphur clusters [20,21].

The fact that mutations in Esx-3 led to resistance to multiple chemical scaffolds suggests that this is a common mechanism of resistance. Since we observed high-level resistance for several classes, we conclude that this mechanism would be clinically relevant and should be further investigated.

## Supplementary material

10.1099/mic.0.001625 Table S1.

## Abbreviations

AMT: aminothiazoles
8HQ: 8-hydroxyquinolines
MOT: morpholino thiophenes
NOA: nitazoxanides
PAI: phenylalkylimidazoles
PHY: phenylhydrazones
TFMP: trifluoromethyl pyrimidinones
